# Exploring the eHealth literacy and related influencing factors in patients after lung cancer surgery: A latent profile analysis

**DOI:** 10.1016/j.apjon.2025.100818

**Published:** 2025-11-11

**Authors:** Yuna Cheng, Yiqing Luo, Xinxing Ju, Jie Yang, Xiaoxin Liu

**Affiliations:** a Department of Oncology Surgery, Shanghai Chest Hospital, School of Nursing Shanghai Jiao Tong University, Shanghai, China; b Nursing Department, Shanghai Chest Hospital, School of Nursing Shanghai Jiao Tong University, Shanghai, China; c Department of Intensive Care Unit, Shanghai Chest Hospital, School of Nursing Shanghai Jiao Tong University, Shanghai, China

**Keywords:** Latent profile analysis, Electronic health literacy, Patients with lung cancer, Self-management, Social support, The transactional model of eHealth literacy

## Abstract

**Objective:**

This study aimed to clarify the subtypes of electronic health literacy among patients with lung cancer surgery and explore the factors affecting profile membership.

**Methods:**

A cross-sectional study utilizing surveys among patients who underwent lung cancer surgery (*n* ​= ​354). Patients completed the general demographic questionnaire, eHealth literacy scale, strategies used by people to promote health scale, perceived social support scale, and functional assessment of cancer therapy lung cancer scale. Data analyses involved latent profile analysis, variance analysis, Chi-square tests, and multivariate logistic regression.

**Results:**

A total of 354 valid questionnaires were collected and categorized into three latent classes based on eHealth literacy levels among post-operative patients with lung cancer: “Low eHealth Literacy,” “Moderate eHealth Literacy,” and “High eHealth Literacy”. Each profile exhibited distinct characteristics representative of the different levels of eHealth literacy among these patients. Factors such as age, educational attainment, occupation type, monthly household income, presence of chronic diseases, daily use of smart devices, frequency of health information searches, variety of eHealth information sources, self-management efficacy, and levels of social support were identified as influencing the eHealth literacy of postoperative patients with lung cancer across these categories.

**Conclusions:**

eHealth literacy among postoperative patients with lung cancer exhibits distinct classification characteristics, with over half falling into low or moderate levels. Identifying the sociodemographic factors and influences affecting different patient groups is crucial for developing internet-based continuity of care measures tailored to the specific needs of these patients.

## Introduction

Lung cancer remains the most common cancer globally, with the highest incidence and mortality rates worldwide.^1^ Surgical resection is the primary curative treatment; however, patients often experience decreased exercise tolerance, impaired pulmonary function, and a high symptom burden postoperatively, with full recovery typically requiring 1–3 months or longer.^2^ After discharge, patients must also manage multiple challenges independently, including treatment decision-making, symptom management, recurrence monitoring, and lifestyle adjustments, highlighting a pressing need for continued care.^3^

In this context, digital health platforms have shown considerable potential in health promotion, treatment adherence management, and remote monitoring.^4^ Yet, the extent to which patients benefit from these interventions largely depends on their eHealth literacy—defined as the ability to seek, understand, evaluate, and apply health information from electronic sources to address health problems.^5^ Although the lung cancer population is increasingly younger, studies indicate that eHealth literacy remains generally inadequate,^6^^,^^7^ creating a notable gap in the context of rapidly evolving digital health landscapes. Thus, investigating eHealth literacy and its determinants in this population is essential.

Most existing studies have adopted a variable-centered approach, which can only reveal average relationships between factors and fails to identify heterogeneous subgroups within the patient population. Understanding such individual-level variation is critical for developing targeted interventions; however, studies applying a person-centered method, such as latent profile analysis ​ (LPA), are still lacking among postoperative patients with lung cancer.

To better understand this heterogeneity, the present study employs the Transactional Model of eHealth Literacy (TMeHL) as its theoretical framework.^8^ This model posits that eHealth literacy emerges from dynamic interactions between users and digital environments, influenced by both task-oriented and user-oriented factors. User-oriented factors reflect an individual's intrinsic motivation, resources, and competencies within digital health contexts and represent key theoretical dimensions underlying group differences in eHealth literacy. Grounded in the TMeHL framework and existing empirical evidence, this study focuses on three core user-oriented factors—self-management efficacy, social support, and self-perceived health status—and proposes that these may drive potential subgroup differences in eHealth literacy among postoperative patients with lung cancer.

Therefore, this study aims to identify latent profiles of eHealth literacy in patients after lung cancer surgery and to examine the characteristics and influencing factors of each profile, thereby informing the development of targeted clinical interventions.

### Postoperative recovery challenges and unmet needs in patients with lung cancer

While enhanced recovery after surgery (ERAS) pathways have shortened hospital stays for patients with lung cancer, they also lead to earlier discharge—often during the initial or middle stages of recovery rather than the later phase. Invasive surgical trauma results in significantly reduced exercise tolerance within the first week, impaired pulmonary function and physical performance by the second week, and diminished activities of daily living even up to four weeks postoperatively.^3^ The prevalence of postoperative symptoms ranges from 48% to 79%,^9^ and a return to preoperative activity levels generally requires 1–3 months or longer.^10^ After discharge, patients face multiple challenges, including making subsequent treatment decisions, managing cancer-related symptoms and side effects, monitoring for disease recurrence, and adapting to healthier lifestyles.

### Applications and limitations of eHealth in lung cancer care

Against this background, internet-mediated platforms—such as health information portals, mobile applications, and WeChat mini-programs—offer promising solutions to address these care gaps. These tools demonstrate considerable potential in supporting postoperative health promotion, improving treatment adherence, enabling remote monitoring, and facilitating clinical decision-making.^4^ However, the extent to which patients benefit equitably from such digital interventions depends heavily on their eHealth literacy. Patients with higher eHealth literacy are better able to utilize online resources, mobile apps, and other digital tools to access disease-related information, rehabilitation guidance, and lifestyle advice, thereby obtaining ongoing support through digital channels.^11^ In addition, eHealth literacy may influence patients’ psychological well-being,^12^ as online support groups, mental health applications, and other digital resources can provide emotional support and coping strategies.

Although the lung cancer population is increasingly younger and thus considered likely to benefit from digital health tools, a study by Milne et al.^7^ revealed that patients generally exhibit low eHealth literacy, influenced by factors such as educational attainment and regional health care development. This creates a mismatch with the rapidly evolving digital health landscape.^13^ A cross-sectional study by Yaman-Çelik and Durmaz-Edeer^6^ among thoracic surgery patients further confirmed only moderate levels of eHealth literacy, which hinders their ability to access and utilize electronic health information effectively. This “digital divide” may be particularly pronounced in certain sociocultural contexts. For instance, in China and other Asian regions, despite growing internet penetration, older, less-educated, and rural patients often face significant barriers in digital skills, access channels, and information discrimination, limiting equitable access to digital health resources.^7^

### Theoretical framework and methodological rationale

Existing research on eHealth literacy has predominantly focused on general chronic disease populations, with a notable lack of studies examining the potential subtypes and their influencing factors specifically among postoperative lung cancer patients. Most previous studies have adopted a variable-centered approach, which overlooks individual heterogeneity and fails to identify distinct subgroups formed by diverse skill sets, cognitive patterns, and behavioral profiles. To effectively identify homogeneous patient subgroups that differ across these dimensions, this study employs LPA, a person-centered modern statistical method. LPA^14^ can identify underlying, unobservable categories based on patients’ response patterns across multiple eHealth literacy indicators, thereby revealing the true heterogeneous structure of the patient population.

**Fig. 1:**
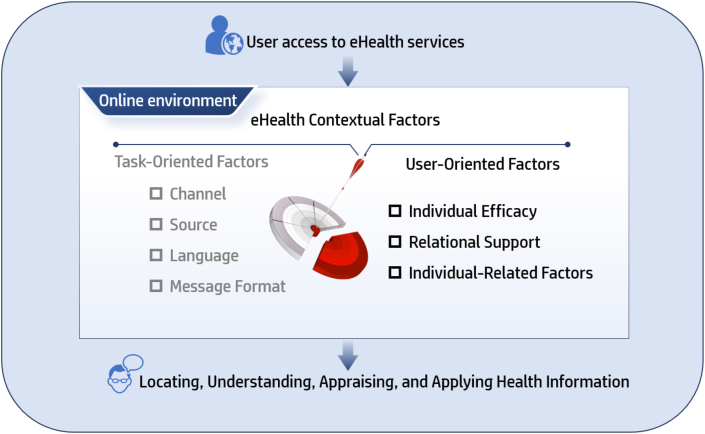
Conceptual model diagram.

To better understand and address these challenges, this study uses the TMeHL as its core theoretical framework. This model moves beyond earlier perspectives that focused solely on information acquisition skills, proposing instead that eHealth literacy emerges from dynamic interactions between user-oriented and task-oriented factors in digital environments. According to this theory,^8^ both task-oriented and user-oriented factors jointly influence users' ability to locate, comprehend, communicate, and evaluate health information in online settings when utilizing eHealth services. User-oriented factors directly reflect an individual's intrinsic motivation, resources, and capability foundations within digital health environments, serving as key theoretical dimensions explaining group heterogeneity in eHealth literacy. These include technological factors (such as self-efficacy and technology use preferences), relational support (support received or perceived social norms when using eHealth services), and personal relevant factors. This framework provides clear guidance for identifying key influencing factors in this study, which include: (1) self-management efficacy^15^^,^^16^—patients' confidence in managing their health information and behaviors, where individuals with higher self-management efficacy show greater propensity to actively explore and apply digital health tools; (2) social support^17^—emotional and material support from family, friends, and the community, serving as crucial external drivers for patients to access digital resources and learn new skills; and (3) self-perceived health status^18^^,^^19^—patients' subjective evaluation of their own health, directly influencing their motivation and ability to seek and utilize online health information. The detailed results are presented in Fig. 1.

Therefore, based on the TMeHL framework, we hypothesize that self-management efficacy, social support, and self-perceived health status are key user-oriented factors influencing eHealth literacy among postoperative lung cancer patients, with these variables constituting personal elements within the user-oriented dimension.

Building on previous research, we aim to further identify the latent categories of eHealth literacy among post-operative lung cancer patients and investigate the characteristics of patients within each category to enhance the specificity of subsequent clinical practices.

## Methods

### Design

A single-center, cross-sectional, descriptive study was conducted using a convenience sampling method. Participants were recruited from the Department of Thoracic Surgery at Shanghai Chest Hospital between April and July 2024. Although convenience sampling may limit the generalizability of findings to other health care settings or regional populations, Shanghai Chest Hospital—as a regional referral center performing over 12,000 thoracic surgeries annually—treats a patient population that is representative in terms of disease characteristics, surgical approaches, and postoperative management. Thus, the sample reasonably reflects the profile of patients with lung cancer after surgery in large specialized medical centers in China.

### Participants

The inclusion criteria for patients were as follows: (1) diagnosed with non-small cell lung cancer without distant metastasis or other tumors, and having undergone surgery with curative intent; (2) at least 18 years of age; (3) fully informed about their diagnosis and treatment; and (4) willing to participate in the study and sign an informed consent form. The exclusion criteria were: (1) cognitive impairment affecting comprehension or self-expression; (2) presence of psychiatric disorders or cognitive dysfunction.

### Sample size

A total of 375 questionnaires were distributed, with 354 valid responses retained. According to empirical studies on sample size adequacy,^20^^,^^21^ a sample size exceeding 300 provides sufficient statistical power to stably identify and compare 3–4 latent profiles under conditions of moderate factor loadings and class separation. Thus, the sample size in this study is adequate for exploring the latent class structure of eHealth literacy among postoperative lung cancer patients. Following the study, a post hoc power analysis was conducted based on the results obtained from 354 participants, revealing that the study's power is 92% at a medium effect size and a 95% confidence level.

### Data collection

The survey was conducted using paper-based questionnaires, which were distributed and collected on the day of the patients' discharge. Three data collectors were uniformly trained in questionnaire distribution, completion requirements, and the use of standardized instructions. Before completing the questionnaire, patients were informed of the study's purpose and asked to sign a written consent form. Patients who provided informed consent could fill out the questionnaire independently, while those unable to do so had the questions read aloud by the researchers, who then recorded their verbal responses. All participants received the same instructions when the questionnaires were distributed. Participant anonymity was strictly maintained, and the data were securely stored. To ensure data quality and minimize potential interviewer bias during survey administration, a standardized protocol was strictly followed. When questionnaires were read aloud to participants at their request, all research staff were trained to deliver the items verbatim in a neutral manner, without providing additional explanations, emphasis, or interpretation. This approach maintained consistency across all data collectors and settings.

### Measures

#### Demographic questionnaire

The demographic questionnaire was developed by the researchers and includes information on age, gender, educational level, marital status, occupation type, monthly household income, and the presence of chronic diseases. Additionally, the questionnaire gathers data on patients' daily usage of smart devices, frequency of health and disease information searches, and preferred sources for health information.

#### eHealth literacy scales, eHEALS

The scale^22^ originally developed by Norman and Skinner^23^ and later translated into Chinese by Guo, comprises three dimensions: ability to access online health information and services, evaluative skills, and decision-making capacity, with a total of eight items. It uses a 5-point Likert scale, ranging from 1 (“strongly disagree”) to 5 (“strongly agree”), yielding a total score between 8 and 40. The scale's Cronbach's *α* coefficient is 0.880, indicating high internal consistency.

#### Strategies used by people to promote health, SUPPH

The scale developed by Lev et al.^24^ and translated into Chinese by Zhou et al.,^25^ is designed to assess personal self-management efficacy. The scale has been validated for reliability and validity and consists of 28 items across three dimensions: stress management, decision-making, and positive attitude. It uses a 5-point Likert scale, ranging from 1 (“not confident”) to 5 (“very confident”). The total score ranges from 28 to 140, with higher scores indicating greater confidence in managing one's condition post-lung cancer surgery. The overall Cronbach's *α* coefficient for the scale is 0.970, with coefficients for individual dimensions ranging from 0.849 to 0.959.

#### Perceived social support scale, PSSS

The scale, originally developed by Zimet^26^ and later translated into Chinese by Jiang,^27^ is used to measure perceived social support across multiple dimensions. It includes 12 items divided into three dimensions: family support, friend support, and other support. The scale employs a 7-point rating system, with total scores ranging from 12 to 84, with higher scores reflecting greater perceived social support. The Cronbach's *α* coefficient for the overall scale and its dimensions ranges from 0.85 to 0.91, indicating strong internal consistency.

#### Functional assessment of cancer therapy lung cancer, FACT-L

The scale, developed by Cella et al.^28^ and translated into Chinese by Wan et al.,^29^ is composed of a generic cancer treatment module and a lung cancer-specific module, designed to assess the quality of life in patients with lung cancer. The scale includes 36 items across five dimensions: physical well-being, social/family well-being, functional well-being, emotional well-being, and additional concerns. It uses a 5-point Likert scale, with scores ranging from 0 (“not at all”) to 4 (“very much”) for positively worded items, while negatively worded items are reverse scored. The total score ranges from 0 to 144, with higher scores indicating better quality of life. The scale's reliability has been validated, with a Cronbach's *α* coefficient of 0.805.

### Data analysis

LPA was conducted using Mplus 8.3 statistical software, with the average scores of the eight items on the eHEALS scale (continuous variables) serving as observed variables for post-operative lung cancer patients. The analysis began with an initial model assuming a single latent class, and additional classes were incrementally added. Model comparisons were made based on fit indices to determine the optimal number of latent classes. Commonly used fit indices include: (i) model evaluation indices—Akaike information criterion (AIC), Bayesian information criterion (BIC), sample size-adjusted BIC (aBIC), and entropy; (ii) model comparison indices—Lo-Mendell-Rubin adjusted likelihood ratio test (LMR) and bootstrap likelihood ratio test (BLRT). AIC, BIC, and aBIC evaluate model fit by comparing the actual values with the expected values, with lower values indicating better model fit. The entropy index, ranging from 0 to 1, reflects classification accuracy, with values closer to 1 indicating more precise classification. LMR and BLRT compare the fit of different latent profile models, with a significant p-value indicating that a model with k classes fits better than a model with k-1 classes. This study employed full information maximum likelihood (FIML) to handle missing data. In subsequent between-group comparisons, we applied the Bonferroni correction to adjust the results of LSD post hoc tests, thereby controlling the inflation of type I error.

Statistical descriptions and analyses were performed using SPSS 27.0 software. Continuous variables were expressed as means and standard deviations, while categorical and ordinal variables were presented as frequencies and percentages. In the univariate analysis of latent profiles of eHealth literacy among post-operative lung cancer patients, one-way ANOVA was used for continuous variables, the chi-square test for unordered categorical variables, and the Kruskal–Wallis H test for ordered categorical variables. The final profile results were used as dependent variables, and multinomial logistic regression was conducted to analyze the factors influencing the latent classes of eHealth literacy among post-operative lung cancer patients. The significance level was set at *α* ​= ​0.05.

### Validity and reliability

The psychometric properties of the measurement instruments have been described above.

## Results

### Participants’ characteristics

A total of 375 questionnaires were distributed, with 354 valid responses collected, resulting in an effective response rate of 94.40%. Among the 375 participants, 3 withdrew from the study; 7 questionnaires were incomplete; and 11 were deemed invalid due to uniform responses across all items or subscales. A comparison of basic demographic characteristics was performed between the complete-case group (*n* ​= ​354) and the missing-data group (*n* ​= ​21). The statistical analysis revealed no significant differences in any of the compared indicators between the two groups (all *P* ​> ​0.05). The average age of participants was 58.10 years (SD ​= ​12.54, range 19–86 years). Detailed demographic information is provided in Table 1.

**Table 1:** Demographic profiles of the participants (*N* ​= ​354).

Variable	*n*	%
**Sex**		
Male	156	44.1
Female	198	55.9
**Age (Mean ​± ​SD, years)**	58.10 ​± ​12.54	
**Education levels**		
High school and below	246	69.5
junior College and above	108	30.5
**Marital status**		
Unmarried	12	3.4
Married	320	90.4
Widowed or divorced	22	6.2
**Occupation**		
Personnel of enterprises and institutions	77	21.7
Self-employed or freelancer	33	9.3
Factory workers or Farmer	191	54.0
others	53	15.0
**Family monthly income (CNY)**		
≤ ​8000	101	28.5
8001–15,000	128	36.2
15,001–20,000	67	18.9
> ​20,000	58	16.4
**Comorbid chronic diseases**		
Yes	115	32.5
No	239	67.5
**Electronic information inquiry frequency**		
Never	61	17.3
Occasionally	130	36.7
Sometimes	102	28.8
Often	61	17.3
**Daily usage time of electronic devices**		
< ​1 hour	65	18.4
1–3 hours	118	33.3
3–5 hours	90	25.4
> ​5 hours	81	22.9
**Types of electronic information inquiry channels**		
0-1	150	42.4
≥ ​2	204	57.6

SD, standard deviation.

### Description statistic and correlations

eHealth literacy among post-operative lung cancer patients is positively correlated with self-management efficacy, social support, and quality of life. Table 2 presents the descriptive statistics and correlations between eHealth literacy, self-management efficacy, social support, and quality of life levels, as well as the scores for each dimension.

**Table 2:** Descriptive statistics and correlations between electronic health literacy, Self-management efficacy, social support and quality of life.

Scale	Mean	SD	1	2	3	4	5	6	7	8	9	10	11	12	13	14	15	16	17	18
**1 Electronic health literacy**	25.88	9.28	1																	
2 Application ability	16.99	5.94	0.982^a^	1																
3 Judgment ability	5.59	2.53	0.930^a^	0.856^a^	1															
4 Decision-making ability	3.30	1.24	0.881^a^	0.817^a^	0.823^a^	1														
**5 Self-management efficacy**	92.80	23.41	0.432^a^	0.421^a^	0.409^a^	0.381^a^	1													
6 Self-decompression	31.65	8.93	0.437^a^	0.429^a^	0.408^a^	0.385^a^	0.961^a^	1												
7 Self-decision	10.08	2.82	0.396^a^	0.390^a^	0.374^a^	0.332^a^	0.902^a^	0.846^a^	1											
8 Positive attitude	51.00	12.53	0.415^a^	0.404^a^	0.395^a^	0.368^a^	0.984^a^	0.909^a^	0.860^a^	1										
**9 Social support**	67.01	10.93	0.567^a^	0.549^a^	0.546^a^	0.501^a^	0.399^a^	0.372^a^	0.368^a^	0.402^a^	1									
10 Family support	25.00	3.04	0.277^a^	0.246^a^	0.306^a^	0.269^a^	0.277^a^	0.250^a^	0.250^a^	0.278^a^	0.755^a^	1								
11 Friends support	20.94	4.53	0.588^a^	0.576^a^	0.552^a^	0.510^a^	0.394^a^	0.371^a^	0.362^a^	0.400^a^	0.942^a^	0.553^a^	1							
12 Other support	21.07	4.57	0.589^a^	0.578^a^	0.554^a^	0.512^a^	0.379^a^	0.356^a^	0.353^a^	0.379^a^	0.953^a^	0.591^a^	0.893^a^	1						
**13 Quality of life**	105.90	15.72	0.134^b^	0.135^b^	0.127^b^	0.100	0.275^a^	0.301^a^	0.267^a^	0.242^a^	0.079	0.018	0.090	0.088	1					
14 Physiological	23.75	3.08	0.153^a^	0.157^a^	0.135^b^	0.119^b^	0.216^a^	0.208^a^	0.180^a^	0.215^a^	0.085	0.040	0.085	0.093	0.708^a^	1				
15 Society	21.07	3.89	0.183^a^	0.190^a^	0.166^a^	0.122^b^	0.273^a^	0.307^a^	0.250^a^	0.247^a^	0.118^b^	0.054	0.127^b^	0.119^b^	0.743^a^	0.506^a^	1			
16 Emotion	17.27	3.56	0.133∗	0.151^a^	0.100	0.067	0.211^a^	0.233^a^	0.226^a^	0.179^a^	0.097	0.030	0.103	0.108^b^	0.779^a^	0.516^a^	0.635^a^	1		
17 Function	16.12	5.02	0.145^a^	0.145^a^	0.152^a^	0.086	0.310^a^	0.362^a^	0.322^a^	0.254^a^	0.088	0.030	0.083	0.108^b^	0.796^a^	0.500^a^	0.570^a^	0.644^a^	1	
18 Additional attention	27.94	4.05	−0.010	−0.010	−0.013	0.002	0.152^a^	0.143^a^	0.165^a^	0.136^b^	0.000	−0.017	0.007	0.005	0.661^a^	0.494^a^	0.289^a^	0.372^a^	0.414^a^	1

SD, standard deviation.

a *P* ​< ​0.01.

b *P* ​< ​0.05.

### Latent profile analysis

#### Exploratory latent profile analysis

The results indicate that as the number of profiles increased, both AIC and BIC values consistently decreased, suggesting improved model fit. The three-class model demonstrated the best fit, with lower AIC (6243.280), BIC (6374.837), and aBIC (6266.974) values, the highest entropy, and the smallest p-values for the LMR test (< 0.001) and BLRT (< 0.001), indicating statistical significance at the *α* ​= ​0.05 level. Beyond statistical indicators, the three-class model demonstrated optimal clinical interpretability and theoretical coherence. It clearly differentiated three distinct and clinically recognizable subgroups: “high literacy,” “moderate literacy,” and “low literacy.” These subgroups exhibited gradient differences in their ability to acquire, comprehend, and apply eHealth information, which aligns closely with the theoretical expectations of the TMeHL. Furthermore, the three-class model adhered to the principle of parsimony, maintaining strong explanatory power without over-segmentation, thereby facilitating the development of targeted clinical intervention strategies.

**Fig. 2:**
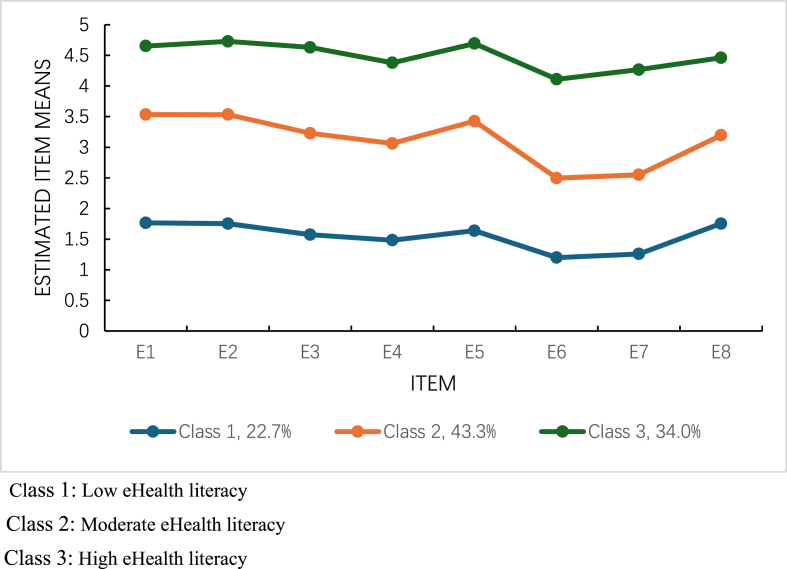
Latent profile of the eHealth literacy among lung cancer patients Class 1: Low eHealth literacy; Class 2: Moderate eHealth literacy; Class 3: High eHealth literacy.

In this three-class model, post-operative lung cancer patients' eHealth literacy was categorized into three latent classes: “Low eHealth Literacy (Class 1)," “Moderate eHealth Literacy (Class 2)," and “High eHealth Literacy (Class 3)," comprising 22.7%, 43.3%, and 34.0% of the sample, respectively. The detailed results are presented in Table 3 and Fig. 2.

**Table 3:** Fit metrics and category probability of Latent Profile Analysis Models.

Model	AIC	BIC	aBIC	LMR	BLRT	Entropy	Category probability				
				*P*-value	*P*-value						
1-profile	9393.356	9455.265	9404.506								
2-profile	7375.050	7471.783	7392.472	0.005	< 0.001	0.938	39.0%/61.0%				
**3-profile**	**6243.280**	**6374.837**	**6266.974**	**< 0.001**	**< 0.001**	**0.965**	**22.7%/34.0%/43.3%**				
4-profile	5800.851	5967.231	5830.817	0.001	< 0.001	0.960	20.6%/34.8%/12.8%/31.8%				
5-profile	5692.708	5893.912	5728.946	0.349	< 0.001	0.925	12.0%/19.0%/15.2%/29.2%/24.6%				

AIC, Akaike information criterion; BIC, Bayesian information criteria; aBIC, adjusted Bayesian information criteria; LMR, Lo–Mendell–Rubin Test; BLRT, Bootstrap Likelihood Ratio Test.

#### Electronic health literacy of patients with different types of lung cancer after surgery

The variance analysis for the total scores and the three dimensions of eHealth literacy showed statistically significant differences across the groups (*P* ​< ​0.001). Further analysis using the Least Significant Difference (LSD) test revealed significant differences in application ability, evaluative ability, and decision-making ability among the three classes, with C1 ​< ​C2 ​< ​C3 (*P* ​< ​0.001). The detailed results are presented in Table 4.

**Table 4:** Electronic health literacy of lung cancer patients after surgery in different categories (*N* ​= ​354).

	*N*	Electronic health literacy	Electronic health literacy- Application ability	Electronic health literacy- Judgment ability	Electronic health literacy- decision-making ability
C1: Low eHealth literacy	80	12.39 ​± ​3.72	8.19 ​± ​2.99	2.45 ​± ​0.76	1.75 ​± ​0.80
C2: Moderate eHealth literacy	153	24.99 ​± ​3.24	16.76 ​± ​2.19	5.04 ​± ​1.39	3.19 ​± ​0.76
C3: High eHealth literacy	121	35.92 ​± ​2.69	23.08 ​± ​1.62	8.37 ​± ​1.14	4.46 ​± ​0.62
*F*		1328.907	1072.315	627.060	1328.907
*P*		< 0.001	< 0.001	< 0.001	< 0.001
LSD		C1 ​< ​C2 <C3	C1 ​< ​C2 ​< ​C3	C1 ​< ​C2 <C3	C1 ​< ​C2 <C3

#### Characteristics of latent profile membership

The univariate analysis revealed statistically significant differences among the three groups in terms of age, educational level, occupation type, monthly household income, presence of chronic diseases, daily usage of smart devices, frequency of health information searches, and the variety of eHealth information sources (*P* ​< ​0.05). There were also statistically significant differences in self-management efficacy and social support levels among the three groups (*P* ​< ​0.05), with the third group exhibiting the highest levels of self-management efficacy and social support. However, differences in self-reported quality of life were not statistically significant (*P* ​> ​0.05). Class 1 comprised patients with “low eHealth literacy,” characterized by a mean age of 66.6 ​± ​6.6 years. The vast majority (97.5%) had a high school education or less, nearly half (48.7%) never actively sought eHealth information, and 46.3% used electronic devices for less than one hour per day. Class 2 represented patients with “moderate eHealth literacy,” with a mean age of 62.48 ​± ​8.45 years. In this group, 39.2% reported 1–3 hours of daily electronic device usage, and more than half (57.5%) utilized two or more channels to access eHealth information. Class 3 consisted of patients with “high eHealth literacy,” averaging 46.92 ​± ​11.56 years in age. Most (71.9%) had completed junior college or higher education, exhibited the most active digital engagement, and the vast majority (85.1%) used two or more channels to seek eHealth information. The detailed results are presented in Table 5.

**Table 5:** The differences in patients’ electronic health literacy latent profiles in demography, self-management efficacy, social support and quality of life (*N* ​= ​354).

Variables	Respondents *n* (%) or Mean ± SD	Low eHealth literacy *n* (%) or Mean ± SD	Moderate eHealth literacy *n* (%) or Mean ± SD	High eHealth literacy *n* (%) or Mean ± SD	*X*^*2*^*/F/H*	*P*
**Sex**						
Male	156 (44.1)	41 (51.2)	65 (42.5)	50 (41.3)	2.200^a^	0.333
Female	198 (55.9)	39 (48.8)	88 (57.5)	71 (58.7)		
**Age (years)**	58.10 ​± ​12.54	66.63 ​± ​6.61	62.48 ​± ​8.95	46.92 ​± ​11.56	132.346^d^	< 0.001
**Education levels**						
High school and below	246 (69.5)	78 (97.5)	134 (87.6)	34 (28.1)	150.577^c^	< 0.001
Junior college and above	108 (30.5)	2 (2.5)	19 (12.4)	87 (71.9)		
**Marital status**						
Unmarried	12 (3.4)	1 (1.3)	2 (1.3)	9 (7.4)	8.470^b^	0.065
Married	320 (90.4)	73 (91.3)	143 (93.5)	104 (86.0)		
Widowed or divorced	22 (6.2)	6 (7.5)	8 (5.2)	8 (6.6)		
**Occupation**						
Personnel of enterprises and institutions	77 (21.7)	6 (7.5)	22 (14.4)	49 (40.5)	59.935^a^	< 0.001
Self-employed or freelancer	33 (9.3)	4 (5.0)	13 (8.5)	16 (13.2)		
Factory workers or Farmer	191 (54.0)	60 (75.0)	97 (63.4)	34 (28.1)		
others	53 (15.0)	10 (12.5)	21 (13.7)	22 (18.2)		
**Family monthly income (**CNY**)**						
≤ 8000	101 (28.5)	43 (53.8)	53 (34.6)	5 (4.2)	67.575^c^	< 0.001
8001–15,000	128 (36.2)	20 (25.0)	62 (40.5)	46 (38.0)		
15,001–20,000	67 (18.9)	10 (12.5)	22 (14.4)	35 (28.9)		
> 20,000	58 (16.4)	7 (8.8)	16 (10.5)	35 (28.9)		
**Comorbid chronic diseases**						
Yes	115 (32.5)	37 (46.3)	53 (34.6)	25 (20.7)	14.948^a^	< 0.001
No	239 (67.5)	43 (53.8)	100 (65.4)	96 (79.3)		
**Electronic information inquiry frequency**						
Never	61 (17.2)	39 (48.8)	18 (11.8)	4 (3.3)	81.450^c^	< 0.001
Occasionally	130 (36.8)	30 (37.5)	65 (42.4)	35 (28.9)		
Sometimes	102 (28.8)	8 (10.0)	48 (31.4)	46 (38.0)		
Often	61 (17.2)	3 (3.8)	22 (14.4)	36 (29.8)		
**Daily usage time of electronic devices**						
< 1 hour	65 (18.4)	37 (46.3)	26 (17.0)	2 (1.7)	70.585^c^	< 0.001
1–3 hours	118 (33.3)	24 (30.0)	60 (39.2)	34 (28.1)		
3–5 hours	90 (25.4)	11 (13.8)	45 (29.4)	34 (28.1)		
> 5 hours	81 (22.9)	8 (10.0)	22 (14.4)	51 (42.1)		
**Types of electronic information inquiry channels**						
0-1	150 (42.4)	67 (83.8)	65 (42.5)	18 (14.9)	93.294^c^	< 0.001
≥ 2	204 (57.6)	13 (16.3)	88 (57.5)	103 (85.1)		
**Self-management efficacy**	92.80 ​± ​23.407	77.60 ​± ​19.79	92.16 ​± ​23.12	103.66 ​± ​20.08	35.869^d^	< 0.001
Self-decompression	31.65 ​± ​8.93	25.95 ​± ​8.09	31.28 ​± ​8.63	35.88 ​± ​7.56	35.947^d^	< 0.001
Self-decision	10.08 ​± ​2.82	8.50 ​± ​2.54	9.94 ​± ​2.73	11.31 ​± ​2.54	28.170^d^	< 0.001
Positive attitude	51.00 ​± ​12.53	42.85 ​± ​10.49	50.94 ​± ​12.54	56.47 ​± ​10.75	33.761^d^	< 0.001
**Social support**	67.01 ​± ​10.93	56.69 ​± ​8.97	67.02 ​± ​9.45	73.83 ​± ​8.20	88.821^d^	< 0.001
Family support	25.00 ​± ​3.05	23.83 ​± ​3.12	24.82 ​± ​3.11	26.01 ​± ​2.58	13.803^d^	< 0.001
Friends support	20.94 ​± ​4.53	16.41 ​± ​3.64	21.08 ​± ​3.81	23.75 ​± ​3.40	98.383^d^	< 0.001
Other support	21.07 ​± ​4.57	16.45 ​± ​3.84	21.12 ​± ​3.87	24.07 ​± ​3.09	107.248^d^	< 0.001
**Quality of life**	105.90 ​± ​15.72	103.20 ​± ​15.75	105.32 ​± ​14.76	108.41 ​± ​16.63	2.859^d^	0.059
Physiological	23.75 ​± ​3.08	23.23 ​± ​3.58	23.59 ​± ​2.85	24.29 ​± ​2.93	3.276^d^	0.039
Society	21.07 ​± ​3.89	20.15 ​± ​3.84	20.80 ​± ​3.96	22.02 ​± ​3.65	6.464^d^	0.002
Emotion	17.27 ​± ​3.56	16.27 ​± ​3.71	17.41 ​± ​3.59	17.75 ​± ​3.32	4.430^d^	0.013
Function	16.12 ​± ​5.02	15.50 ​± ​5.11	15.55 ​± ​4.49	17.24 ​± ​5.43	4.701^d^	0.010
Additional attention	27.94 ​± ​4.05	28.05 ​± ​4.23	27.97 ​± ​4.17	27.81 ​± ​3.78	0.097^d^	0.908

a Chi−square tests.

b Fisher exact test.

c Kruskal–Wallis *H* test.

d The one-way ANOVA.

#### Predictors of latent profile membership

Based on this analysis, a multinomial logistic regression was conducted to verify the factors influencing eHealth literacy across the three latent profiles, using the high eHealth literacy group as the reference. As shown in Table 6, compared to the high eHealth literacy group, post-operative lung cancer patients in the low eHealth literacy group were more likely to use only one or fewer information search channels. Additionally, patients in both the low and moderate eHealth literacy groups were more likely to be older and have an education level below high school. Both groups also exhibited lower levels of self-management efficacy and social support.

**Table 6:** Multinomial logistic regression analysis of potential categories of lung cancer patients’ eHealth literacy.

Variables	Low eHealth literacy VS High eHealth literacy						Moderate eHealth literacy VS High eHealth literacy					
	β	SE	Wald x^2^	*P*	OR	95% CI	β	SE	Wald x^2^	*P*	OR	95% CI
Constants	−0.909	3.789	0.058	0.810	–	–	−4.285	2.686	2.545	0.111	–	–
**Age (years)**	**0.203**	**0.039**	**26.785**	**0.000**^a^	**1.225**	**1.134, 1.322**	**0.166**	**0.029**	**31.744**	**0.000**	**1.180**	**1.114, 1.250**
**Education levels**										a		
**High school and below**	**3.976**	**1.195**	**11.064**	**0.001**^a^	**53.307**	**5.120, 554.985**	**2.346**	**0.601**	**15.229**	**0.000**^a^	**10.447**	**3.215, 33.945**
Junior college and above (refer)												
**Occupation**												
Personnel of enterprises and institutions	−0.297	1.154	0.066	0.797	0.743	0.077, 7.127	−0.196	0.760	0.067	0.796	0.822	0.185, 3.645
Self-employed or freelancer	−1.360	1.194	1.299	0.254	0.257	0.025, 2.662	−0.462	0.798	0.336	0.562	0.630	0.132, 3.008
Factory workers or Farmer	−0.046	0.821	0.003	0.955	0.955	0.191, 4.769	−0.027	0.593	0.002	0.964	0.974	0.304, 3.115
Others (refer)												
**Family monthly income (CNY)**												
≤ 8000	0.812	1.115	0.530	0.466	2.252	0.253, 20.013	0.844	0.804	1.102	0.294	2.326	0.481, 11.243
8001–15,000	−1.202	1.036	1.345	0.246	0.301	0.039, 2.291	−0.699	0.642	1.187	0.276	0.497	0.141, 1.749
15,001–20,000	−1.372	1.110	1.527	0.217	0.253	0.029, 2.235	−0.864	0.706	1.496	0.221	0.422	0.106, 1.682
> 20,000 (refer)												
**Comorbid chronic diseases**												
Yes	0.183	0.600	0.093	0.760	1.201	0.370, 3.895	0.606	0.472	1.645	0.200	1.833	0.726, 4.627
No (refer)												
**Electronic information inquiry frequency**												
Never	0.615	1.341	0.210	0.646	1.850	0.134, 25.623	−0.813	1.061	0.587	0.443	0.443	0.055, 3.548
Occasionally	0.767	0.994	0.596	0.440	2.154	0.307, 15.111	0.187	0.582	0.103	0.748	1.205	0.386, 3.768
Sometimes	1.169	1.044	1.254	0.263	3.218	0.416, 24.890	0.778	0.576	1.822	0.177	2.177	0.704, 6.738
Often (refer)												
**Daily usage time of electronic devices**												
< ​1 hour	2.067	1.289	2.572	0.109	7.903	0.632, 98.844	1.502	1.089	1.904	0.168	4.491	0.532, 37.943
1–3 hours	0.422	0.838	0.254	0.614	1.525	0.295, 7.884	0.763	0.558	1.869	0.172	2.145	0.718, 6.405
3–5 hours	0.604	0.871	0.481	0.488	1.830	0.332, 10.96	0.900	0.574	2.459	0.117	2.460	0.799, 7.581
> ​5 hours (refer)												
**Types of electronic information inquiry channels**												
0-1	**1.654**	**0.676**	**5.984**	**0.014**^a^	**5.228**	**1.389, 19.675**	0.888	0.515	2.972	0.085	2.431	0.886, 6.674
≥ ​2 (refer)												
**Self-management efficacy**	**−0.042**	**0.013**	**10.205**	**0.001**^a^	**0.958**	**0.934, 0.984**	**−0.021**	**0.010**	**4.382**	**0.036**^a^	**0.979**	**0.959, 0.999**
**Social support**	**−0.186**	**0.035**	**29.035**	**0.000**^a^	**0.830**	**0.775, 0.888**	**−0.077**	**0.027**	**7.831**	**0.005**^a^	**0.926**	**0.878, 0.977**

Refer, Reference group; SE, Standard Error; OR, odds ratio; CI, confidence interval.

a Significant at the 0.05 level.

Multinomial logistic regression was performed to identify factors distinguishing the eHealth literacy profiles, with the high-literacy class (Class 3) as reference. Age and education emerged as consistent demographic predictors across comparisons. Each additional year of age significantly increased the odds of belonging to Class 1 (OR ​= ​1.225, 95% CI: 1.134–1.322) versus Class 3. Patients with high school education or below had substantially higher odds of being in Class 1 (OR ​= ​53.307, 95% CI: 5.120–554.985) or Class 2 (OR ​= ​10.447, 95% CI: 3.215–33.945) compared to Class 3.

Among digital behavior factors, using only 0–1 information channels significantly increased the likelihood of Class 1 membership (OR ​= ​5.228, 95% CI: 1.389–19.675). Psychosocial factors demonstrated strong discriminative power: each unit increase in self-management efficacy reduced the odds of Class 1 membership by 4.2% (OR ​= ​0.958, 95% CI: 0.934–0.984), while each unit increase in social support reduced these odds by 17.0% (OR ​= ​0.830, 95% CI: 0.775–0.888).

## Discussion

### Main findings

Latent profile analysis revealed three distinct classes of eHealth literacy among postoperative lung cancer patients: “low eHealth literacy” (22.7%), “moderate eHealth literacy” (43.3%), and “high eHealth literacy” (34.0%). Key factors associated with class membership included age, educational level, occupation, monthly household income, comorbidity status, daily electronic device usage time, health information-seeking frequency, diversity of eHealth information channels, self-management efficacy, and social support.

Understanding the categories and influencing factors of eHealth literacy among post-operative lung cancer patients helps clarify their health management needs and challenges, and provides a scientific basis for designing targeted clinical interventions and health education strategies. Using latent profile analysis, we identified significant variability in eHealth literacy within this population, confirming hypothesis (a). Three distinct profiles emerged: Low eHealth Literacy (Class 1, 22.7%), Moderate eHealth Literacy (Class 2, 43.3%), and High eHealth Literacy (Class 3, 34.0%). The limited eHealth literacy in Class 1 underscores the need for tailored interventions to address specific barriers. Class 2, representing the majority of patients, indicates that moderate eHealth literacy is the most common level in this Chinese post-operative lung cancer cohort, a finding consistent with previous research.^6^ In this study, patients with lung cancer exhibited moderately higher eHealth literacy scores compared to a Turkish cancer cohort.^30^ This discrepancy may be partly explained by the higher proportion of participants with at least a high school education in our sample. Moreover, as all patients had recently undergone surgery and were in an active treatment and recovery phase, they likely experienced increased demand for specific guidance on rehabilitation, recurrence monitoring, and symptom management. These pressing and concrete needs may have encouraged more proactive online health information-seeking behaviors.

Nevertheless, as shown in the three-class model, the “evaluative ability” dimension consistently scored lower than other dimensions across all patient subgroups—including those with high eHealth literacy. This pattern may stem from exposure to fragmented and often conflicting health information from diverse sources such as social media, health websites, online forums, and academic databases. The variability and unreliability of these sources complicate patients' ability to critically evaluate information, especially when it involves complex medical terminology and treatment regimens.^31^ Consequently, patients without medical training may be more vulnerable to misinformation and face greater difficulty in assessing the scientific credibility and reliability of health-related content. Additionally, the rapid proliferation of digital health information and its highly variable quality present significant challenges to patients’ judgment. The online environment contains a mixture of professional medical content, commercial promotions, and unverified personal anecdotes. Such substantial heterogeneity in information quality demands robust scientific literacy and critical thinking skills for effective discrimination.

Differences in sociodemographic characteristics among the subgroups supported hypothesis (b). Patients in Class 1 were generally older, with 97.5% having a high school education or less, lower household incomes, and a higher proportion engaged in manual or agricultural labor (75%) compared to other groups. These characteristics underscore the particular challenges this subgroup faces in utilizing and evaluating eHealth information. Lower educational attainment may limit their capacity to comprehend health-related content—including medical terminology—and impair their ability to effectively use digital tools for obtaining health information.^30^ Moreover, lower income often correlates with constrained access to resources—such as limited availability of health information and the inability to acquire or maintain smart devices.^32^ The high representation of individuals in agriculture or manual labor in this group further suggests reduced exposure to technology and digital information, compounding these barriers. Notably, nearly half (46.3%) of the patients reported using smart devices for less than one hour per day and relied on limited channels for accessing eHealth information. This low engagement with technology likely restricts their ability to effectively use online health resources.^33^ Consequently, these patients may depend more heavily on traditional information sources, such as word-of-mouth or direct communication with health care providers, rather than proactive online information-seeking. The complexity and variety of contemporary medical information require competencies in searching, filtering, and evaluating content—skills this subgroup often lacks, resulting in underutilization of available resources. An additional noteworthy finding is that 46.3% of patients with chronic diseases also demonstrated low eHealth literacy. This may reflect the increased complexity of managing comorbid conditions, which—along with advanced age—could further impede their ability to use eHealth tools effectively.

To advance the understanding of eHealth literacy complexity, this study employs the TMeHL as its conceptual foundation.^8^ This framework moves beyond earlier models that primarily emphasized information acquisition skills by conceptualizing eHealth literacy as a dynamic interaction between user-oriented and task-oriented factors within digital environments. In this study, user-oriented factors provided the theoretical basis for identifying key personal determinants—such as self-management efficacy, social support, and self-perceived health status—that explain subgroup heterogeneity in eHealth literacy profiles. Simultaneously, the task-oriented dimension guided the investigation of how information-seeking behaviors and digital channel usage patterns interact with personal characteristics to shape distinct literacy profiles. This theoretical integration enables a more nuanced analysis of how personal capabilities and digital task characteristics collectively influence patients' ability to locate, comprehend, communicate, and evaluate health information in online settings.

Self-management efficacy influences eHealth literacy through multiple mechanisms. First, it acts as a cognitive driver, enhancing patients' persistence when confronting health information challenges—those with high self-efficacy are more likely to perceive difficulties in searching for eHealth information as surmountable obstacles rather than insurmountable barriers.^34^ Second, it serves as a behavioral translation bridge, enabling patients to convert acquired digital health knowledge into concrete self-management behaviors.^35^ More importantly, self-management efficacy may function as an emotional regulator, buffering frustration in digital health environments and sustaining motivation for continued engagement in eHealth activities.

Social support promotes eHealth literacy through multidimensional pathways. In terms of instrumental support, practical assistance from family members or peers directly reduces technical barriers for patients using digital health tools.^36^ On the emotional support level, a robust support network alleviates anxiety associated with complex health information, fostering a psychological environment conducive to digital skill development.^37^ Crucially, social support may serve as an information filter, helping patients discern information quality through shared experiences and collective decision-making—a process particularly vital in information-saturated digital environments.

However, our study found that although slight variations in self-reported quality of life were observed among the three patient groups, these differences were not statistically significant—contrary to the results reported by Kyaw et al.^18^ This discrepancy may be attributed to several factors. Kyaw et al.‘s study sampled general older adults in Korea, who are likely more motivated to actively seek health information for managing chronic diseases such as hypertension or diabetes. In contrast, our study focused on patients shortly after major lung cancer surgery, whose early postoperative quality of life is often dominated by acute physiological and psychological stressors, including surgical complications, fear of recurrence, and high symptom burden. These pronounced factors may obscure any modest influence of eHealth literacy, higher overall quality of life does not inherently correlate with better digital health access or skills. In summary, our findings underscore the complex relationship between quality of life and eHealth literacy in postoperative lung cancer patients.^38^ These findings suggest that in clinical practice, efforts to improve eHealth literacy among postoperative patients with lung cancer should be integrated into comprehensive rehabilitation programs addressing physical recovery, psychological adaptation, and social support, rather than implemented as isolated interventions.

### Implications for nursing practice and research

Within the framework of information-based medicine, the limited eHealth literacy observed among post-operative patients with lung cancer poses a significant challenge in adapting to contemporary information-driven health care. Existing digital platforms often require systemic improvements and policy support at institutional and managerial levels—changes difficult to achieve rapidly under resource constraints. Therefore, enhancing patients’ eHealth literacy through self-directed and resource-efficient approaches may represent a key strategy to maximize the benefits of digital health tools during rehabilitation.

In clinical practice, these findings suggest integrating eHealth literacy assessment as a routine component of discharge planning. Implementing stratified screening and management using standardized tools would enable prompt identification of patients with low eHealth literacy and facilitate the establishment of tailored intervention profiles. Postoperative follow-up care should incorporate nurse-led digital skills training, emphasizing how to access authoritative health information, navigate hospital-certified platforms, and identify unreliable content. For patients with low eHealth literacy—particularly older adults and those with limited education—development of illustrated educational materials, voice-interactive systems, and community volunteer-assisted digital navigation is recommended.^39^ Those with moderate to high literacy may benefit from advanced self-management support that includes decision aids and intelligent monitoring tools.

Furthermore, enhancing self-management efficacy and establishing family-centered digital support networks are essential for improving eHealth literacy. Future studies should focus on developing multi-media (e.g., short videos, hotline support, and visual guides) and multi-level digital continuing care models. Integrating eHealth literacy training into oncology nursing education would ensure that health care professionals acquire relevant competencies in assessment and patient guidance. The design of informatics-based continuing care strategies should adopt multi-channel, layered approaches^37^ and promote patient-involved co-design in the development of hospital-based health platforms. Such user-centered design will help ensure intuitive interfaces and practical functionality that meet the needs of patients with diverse literacy levels, thereby reducing barriers to health information access and participation in self-management.

### Limitations

This study has several limitations. First, the use of self-reported questionnaires may introduce social desirability and recall biases. Second, although standardized instruments were applied, self-administered measures are susceptible to interpretation-related measurement error. Third, despite the inclusion of multidimensional variables, unmeasured confounders—such as cognitive status, cancer stage, and surgical complexity—could affect the accuracy of the findings. Fourth, the convenience sampling strategy at a single medical center in Shanghai may lead to selection bias and limit the generalizability of the results to other health care settings. Fifth, latent profile analysis relies on statistical assumptions such as local independence, and there remains a possibility of class misclassification. Finally, although the current sample size was adequate for the analyses, studies with larger samples are needed to enhance the stability and generalizability of the model.

## Conclusions

Based on their eHealth literacy levels, post-operative lung cancer patients were classified into three latent categories: Low (22.7%), Moderate (43.3%), and High (34.0%). Each group displayed distinct characteristics reflective of varying eHealth literacy capabilities. Key influencing factors included age, education, occupation, monthly household income, presence of chronic diseases, daily smart device usage, health information-seeking frequency, diversity of eHealth sources, self-management efficacy, and social support. Health care systems could implement routine eHealth literacy screening during the early postoperative follow-up of patients with lung cancer. For patients with low eHealth literacy, care teams should provide simplified multimedia educational materials and guided digital navigation support. Patients with moderate to high eHealth literacy may benefit from structured training to enhance their ability to critically evaluate and apply online health information, thereby maximizing the effectiveness of internet-mediated rehabilitation platforms in the recovery of postoperative lung cancer patients. At the policy level, our findings support the integration of eHealth literacy support into cancer rehabilitation frameworks and encourage the development of linguistically and culturally adapted digital health tools for diverse patient groups. Future longitudinal studies are needed to examine the stability and long-term trajectories of these literacy classes and to explore their predictive value for health outcomes. Additionally, further research should focus on developing artificially intelligent, personalized eHealth literacy interventions tailored to individual patient characteristics.

## CRediT authorship contribution statement

**Cheng Yuna**: Conceptualization, Methodology, Software, Writing- Original draft. **Luo Yiqing**: Data curation, Writing-review and Editing. **Ju Xinxing**: Visualization, Investigation. **Yang Jie**: Supervision, Software, Validation. **Liu Xiaoxin**: Writing-Reviewing and Editing. All authors have read and approved the final manuscript.

## Ethics statement

The study was approved by the Institutional Review Board of the Shanghai Chest Hospital (Approval No. IS24125) and was conducted in accordance with the 1964 Helsinki Declaration and its later amendments or comparable ethical standards. All participants provided written informed consent.

## Data availability statement

The data that support the findings of this study are available on request from the corresponding author, XL. The data are not publicly available due to the reason that the data containing information that could compromise the privacy of research participants.

## Declaration of generative AI and AI-assisted technologies in the writing process

No AI tools/services were used during the preparation of this work.

## Funding

This study was supported by the Nursing Discipline Construction Project of Shanghai Jiao Tong University School of Medicine (Grant No. SJTUHLXK2024); Shanghai 2024 "Science and Technology Innovation Action Plan" Science Popularization Special Project (Grant No. 24DZ2300700); Shanghai Jiao Tong University School of Nursing: Advanced Specialized Nursing Practice Base Program. The funders had no role in considering the study design or in the collection, analysis, interpretation of data, writing of the report, or decision to submit the article for publication.

## Declaration of competing interest

The authors declare no conflict of interest.
